# Teachers' approaches to music performance anxiety management: a systematic review

**DOI:** 10.3389/fpsyg.2023.1205150

**Published:** 2023-07-31

**Authors:** Isabella Mazzarolo, Kim Burwell, Emery Schubert

**Affiliations:** Empirical Musicology Laboratory, School of the Arts & Media, University of New South Wales, Sydney, NSW, Australia

**Keywords:** stage fright, performance pedagogy, music education, music performance, PRISMA, music pedagogy, music studio teaching, instrumental and vocal teaching

## Abstract

Performance anxiety is a widespread issue that can affect musicians across their education and career. It can develop in musicians from a young age leading to short-term and long-term impacts on not only their performance, but also their wellbeing. There is potentially a significant role that music educators hold in the development of their students and how they handle performance anxiety, though it is not clear how, or how often, teachers support their students in this way. Through a PRISMA-based systematic review, this paper explores what is known about the strategies used by music educators to help manage their students' performance anxiety. The paper also discusses the role that instrumental/vocal tutors and school classroom teachers might hold in this area. The findings show that music educators are implementing multiple strategies to assist their students with MPA, with the most common being simulated performance, positive outlook, preparation and breathing. It was found that there is a role for teachers to address MPA management with their students. While some students prefer to receive MPA support from experts in the field of psychology, students still expressed a need to have this support come from their teacher. Though many teachers felt a need for additional training for them to help their students cope with MPA, many of the strategies were found to be multifunctional and embedded into the regular teaching practices or teaching styles of the educator. Although these strategies might be implicit rather than explicit, the findings suggest that music educators could represent a valuable source of support for MPA management.

## 1. Introduction

Performance anxiety is a widespread area of concern that can impact individuals across a range of endeavors, including public speaking, sport, and performing arts including dancing, acting and music. According to Marchant-Haycox and Wilson (1992), amongst the performing arts, it is musicians who are the most affected by performance anxiety. An emphasis on high standards, often subject to intense scrutiny, creates a constant pressure for musicians to meet their own and others' expectations. The effect of a rigorous routine of practicing, rehearsing and performing, can often result in somatic complaints and emotional fatigue, particularly in young musicians (Stoeber and Eismann, 2007). Consequently, ambitious musicians must not only excel in technical skills and musicality, but importantly, must develop stamina in order to endure the physical and mental demands of performance (Sušić, 2018).

There are many studies that explore these issues, investigating potential causes, symptoms, and strategies that musicians use to cope with music performance anxiety (MPA) (Fehm and Schmidt, 2006; Biasutti and Concina, 2014; Kenny et al., 2014). However, there is little research focused on how this knowledge is applied, particularly by music teachers, who are typically, implicitly trusted to prepare the developing musician for a professional career. This review provides an overview of what is known about how various music educators, including instrumental/vocal tutors, school classroom teachers and university teachers, support their students with MPA. The objectives of this review are:

1. To identify what strategies are used by music educators to help manage their students' performance anxiety;
2. To understand whether music educators have a role in the MPA management of their students.

## 2. Literature review

There are two papers already that systematically reviewed the literature on MPA in relation to music education (Blair and van der Sluis, 2022; MacAfee and Comeau, 2022). Blair and van der Sluis (2022) identified interventions across the literature that could be generalized in higher education settings, including mental skills training, cognitive and imagery strategies, performance psychology training, multimodal coping strategies, biofeedback training, heartrate/biofeedback and emotional refocusing, music performance skills courses, acceptance and commitment therapy, meditation, mindfulness, yoga, desensitization, virtual reality exposure, exam scheduling, and expressive writing. However, the researchers acknowledged that many of the interventions tended to rely heavily on specialist training, delivered by expert physicians or psychologists, rather than music educators. The review by Blair and van der Sluis (2022) contributed to what is known about transferring tested interventions to the teaching setting, but as the strategies' applicability to higher education training was limited, further investigation into how music educators commonly implement MPA management strategies in their everyday teaching is still needed.

MacAfee and Comeau (2022) have also reviewed the MPA literature to identify the five most common strategies music teachers use to support young musicians with MPA. Differing from the previous paper, this paper took professional literature into account, such as magazines and newsletter articles written by studio instrumental teachers, as well as scientific literature. The authors found that preparation, open communication, realistic expectations, exposure to performance, and deep breathing were the most common strategies. The paper also included an analysis of semi-structured interviews with five private piano teachers to explore how these teachers described the five identified strategies reported in the literature, which will be examined in the Results and Discussion of this paper. The review by MacAfee and Comeau (2022) focused on how music teachers support young students with MPA; however, given that performance anxiety can affect musicians of all ages, understanding how teachers manage this issue with older students is also necessary and requires further research.

Typically, the role of the teacher in the development of students' performance skills is regarded as important, but their role in the development and management of MPA is unclear (Ryan and Andrews, 2009). Research has shown that musicians can experience MPA across varying points of their musical studies and career. Kaleńska-Rodzaj (2020) found that among musicians aged 9 to 12, of the 45% of participants who experienced MPA, 31% believed it had a negative impact on their performance, and 18% reported helplessness in coping with it. Many studies have also found MPA to be common at university and professional level (Fishbein et al., 1988; Kenny et al., 2004; Sousa et al., 2016), with some research showing that tertiary-level music students experience higher frequencies of MPA than professional musicians (Steptoe and Fidler, 1987; Wesner et al., 1990; Tamborrino, 2001). University students are at the beginning of their professional career and are at a stage of their development where they are likely to receive more criticism than in the past. In addition, higher education includes more musicians, most of whom do not progress to become professional performers. This might reflect the higher frequency of MPA that they experience and might suggest that a failure to manage MPA can obstruct careers as professional musicians before they start (Fehm and Schmidt, 2006; Patston and Osborne, 2016). Whether a student is learning music as an aspiring professional or for leisure, MPA can still be a significant issue impacting their overall wellbeing and enjoyment of performing. The need for support in MPA prevention and management at an early stage, and throughout a musician's education might therefore be crucial to their development as a musician.

Due to the prevalence of MPA among musicians across varying points of their musical studies and career, research has focused on how to alleviate this issue. Studies have shown that social support can play a significant role in managing MPA, particularly from instrumental teachers and peer support within institutional settings (Tahirbegi, 2019). Social support includes various types of psychological assistance, often provided by significant people within the learner's environment such as family, teachers, and peers (Orejudo et al., 2021). However, those who experience severe MPA may not seek help due to a perceived stigma around this problem and shame in admitting that they suffer from MPA. Wesner et al. (1990) reported that only about 15% of undergraduate student musicians affected by MPA seek help. This might make it difficult for educators to be aware of a student suffering MPA, and to identify the need for support. For educators to recognize instances of MPA within their students and to assist in the management of MPA, there must be an understanding of how musicians themselves experience it and their coping tendencies (Tahirbegi, 2019).

The one-to-one tutor may be well placed to assist with MPA management, Kokotsaki and Davidson (2003) suggesting that they have the potential to understand their students' background, experience, and emotional processes. With this knowledge and the dedicated time that they have with each student on a regular basis, they may be in a good position to discuss psychological issues with their students, such as MPA (Mahony et al., 2022). Classroom music teachers and university teachers might hold a similar role in supporting their students with MPA because, according to Mahony et al. (2022), there is an increasing need for educators in this context to help handle their students' mental health needs. At university level, some institutions are recognizing the need to provide MPA intervention courses for their students and research has looked at how to integrate these interventions into course settings (Spahn et al., 2016). Therefore, the education space has made progress in addressing MPA to support student musicians.

This study addresses the above stated objectives through an overview of the role that educators hold in the MPA management of their students. A particular focus on the MPA strategies that are commonly used in day-to-day lessons, as distinct from interventions that could be generalized in higher education settings (Blair and van der Sluis, 2022), will also be examined.

## 3. Method

The approach taken in this paper is a PRISMA (Preferred Reporting Items for Systematic Reviews and Meta-Analyses)-based systematic review (Liberati et al., 2009).

The inclusion criteria used in the review of the literature are listed below:

- *Types of studies:* Articles reporting original research using both quantitative and/or qualitative methodologies such as questionnaires and/or face-to-face and/or online interviews.
- *Types of participants:* Music educators including instrumental/vocal tutors, school classroom teachers and/or university teachers. Undergraduate music students were also included as there were several studies that reported on approaches taken by musicians' teachers to support them with MPA management.
- *Types of intervention:* Articles referring to strategies used to manage MPA in the teaching setting, or the role that educators hold in this area.
- *Language of publication:* Articles published in English.
- *Publication status:* Final and Article in Press.
- *Rigor:* Peer-reviewed articles.
- *Date range of publications:* Articles published over two decades, set as 1 January 2003 to 31 December 2022.

### 3.1. Information sources and study selection

**Figure 1:**
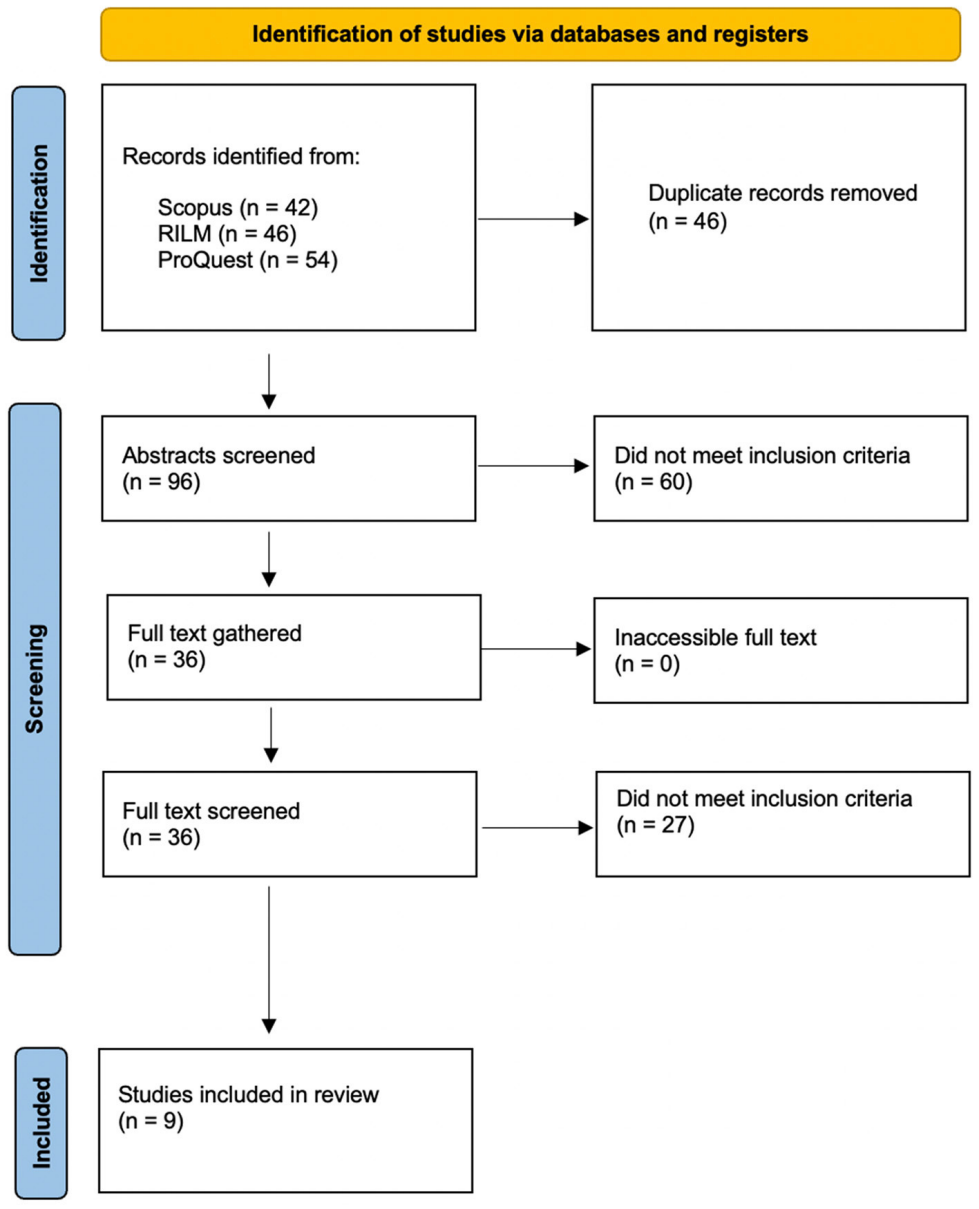
The PRISMA screening process (Page et al., 2021).

Studies were identified by searching electronic databases. This search was applied to Scopus, Répertoire International de Littérature Musicale (RILM) and ProQuest. The following search terms were applied to the title, abstract and keywords across the databases: “music performance anxiety” AND (“teacher” OR “education” OR “pedagogy”). The search provided a total of 96 articles, excluding duplicates (Figure 1). The abstract of each of these articles were examined. Of these, 60 were discarded because they did not meet the inclusion criteria of the intervention type: they did not make reference to teaching strategies used to manage MPA or the role that educators hold in this area. The full text of the remaining 36 articles was examined in more detail. Of these articles, 27 did not meet the inclusion criteria of the intervention type. This left 9 studies that met the inclusion criteria and were included in the review (see Table 1). Of these studies, six referred to strategies used by music educators to manage MPA in the teaching setting (Table 2) and six referred to the role that educators hold in MPA management (Table 3). Figure 1 shows the PRISMA flow diagram (Page et al., 2021).

**Table 1:** Characteristics of articles included in the review.

**References**	**Title**	**Aim**	**Research method**	**Participants**
Cornett and Urhan (2021)	Performance anxiety experiences and coping techniques of Turkish music students and their teachers	To investigate the MPA experiences of musicians in Turkey, including their physical and cognitive symptoms of anxiety, their methods of coping with performance stress, and their perceived need for related resources.	Questionnaire	35 teachers, 230 students
Gill et al. (2022)	Sources of self-efficacy in class and studio music lessons	To understand how music educators foster self-efficacy for performing and enable students to manage the psychological and physiological arousal that accompanies performance situations.	Questionnaire	176 studio music teachers and 128 school music educators
Huang and Yu (2022)	Social support in university music students' coping with performance anxiety: People, strategies and performance situations	To explore the collaborative nature of university music students' MPA coping throughout instrumental/vocal learning and performance preparation.	Semi-structured interviews	99 undergraduate music students
MacAfee and Comeau (2022)	Teacher perspective on music performance anxiety: An exploration of coping strategies used by music teachers	To explore MPA from music teachers' perspectives by identifying and describing common coping strategies teachers use to support students with MPA.	Semi-structured interviews	5 private piano teachers
Moura and Serra (2021)	Listening to teachers' voices: Constructs on music performance anxiety in artistic education	To investigate instrumental teachers' voices to provide a representation of MPA management practices and conceptions.	Semi-structured interviews	4 instrumental tutors (1 keyboard tutor, 1 wind tutor, 2 strings tutors)
Ryan et al. (2021)	Performance preparation, anxiety, and the teacher. Experiences of adolescent pianists	To examine the experiences of adolescent pianists in their private lessons and solo performances in regard to concert preparation and MPA.	Questionnaire	62 adolescent piano students
Sieger (2017)	Music performance anxiety in instrumental music students: A multiple case study of teacher perspectives	To investigate the strategies and methods utilized by middle and high school music teachers to address the MPA experienced by their students.	Multiple case study (interviews and follow up interviews)	3 middle and high school instrumental music teachers
Studer et al. (2011)	Stage fright: Its experience as a problem and coping with it	To assess negative feelings of MPA before performing, the experience of stage fright as a problem, and how closely they are associated with each other.	Questionnaire	190 undergraduate music students
Tahirbegi (2019)	Higher music education students' experiences and management of performance anxiety: A qualitative study	To investigate students' experiences and management of MPA in educational settings.	Semi-structured interviews	10 undergraduate music students

**Table 2:** Strategies identified across the literature.

	**Sieger (2017)**	**Moura and Serra (2021)**	**Gill et al. (2022)**	**Huang and Yu (2022)**	**MacAfee and Comeau (2022)**	**Ryan et al. (2021)**
Breathing	X	X		X	X	X
Diet				X		
Focused attention	X	X	X			X
Imagery			X	X		
Open MPA discussions	X	X	X		X	
Physical activity	X					
Positive outlook	X	X	X	X		X
Pre-performance routines			X			
Preparation	X	X	X		X	X
Psychological performance skills			X			
Repertoire					X	
Safe environment	X	X				
Self-talk			X			
Simulated performance	X	X		X	X	X
Suppression						X
Visualization	X		X	X		

**Table 3:** Teacher and student perceptions of teachers' role in MPA management.

**References**	**Theme**	**Findings**
Cornett and Urhan (2021)	Expert help needed	When asked about resources for MPA management, 20.8% of students would like to work with an expert in the field of MPA management and 33.8% mentioned that psychological support would be useful.
Moura and Serra (2021)	Training required	All 4 teachers said it is part of their role as educators to address MPA, but mentioned they require more training such as practical MPA management projects during university classes and further academic education in this area.
Ryan et al. (2021)	Teachers' role	42% of students said that their teachers provided advice about MPA management, but that much of this advice did not effectively address the issue or provide tangible coping strategies.
Sieger (2017)	Training required	All 3 teachers said that more training is needed in MPA management. One teacher noted they avoid addressing MPA due to the stigma around the topic.
Studer et al. (2011)	Teachers' role, expert help needed	Majority of students (73%) would like to receive support in MPA management from specialists (e.g., psychologists, psychotherapists, or general practitioners), but more than half of the students (56%) would like to receive more information from their university teacher about MPA management.
Tahirbegi (2019)	Teachers' role, expert help needed	All students mentioned the importance of receiving emotional support from their teachers. Some students said they did not feel comfortable discussing MPA with their teachers (exact number of students unknown).

## 4. Results

The full text of the articles that met the research conditions was examined and explicit strategies were noted and coded into relevant themes. For example, articles that mentioned “performance opportunities”, “performance exposure”, “performance practice”, or “playing through repertoire in front of family/friends” were regarded as “simulated performance”; “play for fun”, “mistakes don't matter”, and “you'll be happy you did it” were regarded as “positive outlook”. Coding was completed by author IM and cross-checked with all authors. Information was extracted from each article including: types of studies (questionnaires and/or interviews), aim of the paper, types of participants (types of music educators and/or students), strategies identified and/or viewpoints on the role of addressing MPA. Examining the methods used in the reported studies, it was found that 4 of the studies used questionnaires, while 5 of the studies used semi-structured interviews.

We then examined the strategies used by music educators to help manage their students' performance anxiety. Several strategies were identified across the articles including: preparation (in 5 articles), simulated performance (5), positive outlook (5), breathing (5), focused attention (4), open MPA discussions (4), visualization (3), safe environment (2), imagery (2), repertoire (1), physical activity (1), psychological performance skills (1), pre-performance routines (1), self-talk (1), diet (1), and suppression (1) (see Table 2).

Table 3 provides an overview of the articles which referred to the role that educators hold in MPA management. A range of perspectives was found from both students and teachers, with some students and teachers believing that it is the teacher's role to address MPA management, while other students felt experts in the field such as psychologists, psychotherapists, or general practitioners, would be more suitable. Most teachers believed that they needed additional training in MPA management to support their students in this area.

## 5. Discussion

The reviewed literature offered a broad discussion of teachers' approaches to MPA management. Interestingly, almost all of the articles included in this review were published in the past two years, suggesting that there is an emerging interest in this topic from a pedagogical perspective. An array of strategies used by teachers to support their students with MPA were identified in the papers. The following strategies appeared most frequently, with each mentioned in five of the studies: preparation, simulated performance, positive outlook and breathing. These strategies overlap with the findings from MacAfee and Comeau (2022), who also found that preparation, simulated performance and breathing were the most common MPA strategies used by teachers, along with open communication.

Cognitive strategies emerged as less frequent in the present review which focused on common teaching practices, than in the studies reviewed by Blair and van der Sluis (2022), who focused on interventions that could be generalized to higher education settings. Blair and van der Sluis (2022) defined cognitive strategies as those that include “cognitive psychology and therapeutic interventions using cognitive and imagery strategies to manage or treat MPA” (p. 9). In the study by Gill et al. (2022), fewer than half the studio tutors and classroom teachers used psychological performance skills to combat MPA, and those that did would mostly only do so if the student was already experiencing MPA problems. This further supports a key finding from Blair and van der Sluis (2022), that a significant level of training or support might be required to implement cognitive strategies into regular teaching, as these cognitive strategies are often used by experts in the field of psychology.

In this review and the literature, the terms “strategies”, “MPA management strategies”, “coping skills”, “coping strategies”, and “coping tools” have been most commonly used to refer to the ways in which educators have supported their students with MPA (Moura and Serra, 2021; Gill et al., 2022; Huang and Yu, 2022; MacAfee and Comeau, 2022). Arguably, however, the strategies are multifunctional, in that they enhance both performance skills and MPA management (Huang and Yu, 2022), so it might be more appropriate to identify these strategies as coinciding with the regular teaching practice of the educator, which indirectly influences MPA, rather than explicit strategies. This reflects a finding identified by Huang and Yu (2022), that several students felt their teachers were not aware of their role in supporting their students with MPA management, but rather, they supported their students in an implicit way as the strategies were already embedded into their regular pedagogical process. For example, preparation, which involves task-oriented strategies such as technical drills, mastering specific phrases, slow practice, and repetition (Moura and Serra, 2021), is often a teacher's priority, regardless of their students' MPA situation. In this regard, preparation improves confidence through task mastery, therefore—perhaps incidentally—minimizing MPA (Gill et al., 2022).

Similarly, Moura and Serra (2021) made reference to “MPA management pedagogical practices”, “MPA management strategies”, and the “development of intervention projects”, all of which blur the distinction between MPA management and more general approaches to studio teaching. The authors argued that MPA strategies should not be taught independently to avoid inflaming MPA, aligning with the idea that MPA management is not commonly taught through concern of enhancing it and is clouded by the stigma around the topic (Sieger, 2017). Instead, Moura and Serra (2021) recommended that MPA management strategies should be integrated into performance teaching: into the regular pedagogical practices of the teacher. This was reflected by their findings of teachers who reported mentoring their students in MPA management as part of their role as an educator, integrating it into their lessons through strategic activities such as simulated performance. This prominent strategy can increase self-efficacy when students succeed in relatively low-intensity environments and so experience greater comfort on stage (MacAfee and Comeau, 2022).

Embracing a positive outlook, too, might be regarded more as a teaching style rather than strategy. It includes teachers reacting positively to mistakes and normalizing errors as opportunities for learning (Sieger, 2017; Moura and Serra, 2021). One example was reported by Gill et al. (2022) who highlighted the term “verbal persuasion”, which refers to feedback and encouragement from others, as a strategy for teachers to use to enhance the self-efficacy and therefore, reduce the performance anxiety of students. A similar example came from Moura and Serra (2021), who reported teachers boosting the confidence of their students by highlighting how well prepared they are for their performance. Teachers embracing a positive outlook may help students to adopt this same attitude, increasing their self-efficacy and thereby reducing MPA. Whether teachers embrace a positive outlook might be dependent on whether they foster an encouraging environment and normalize mistakes, rather than being more focused on perfectionism and mastery.

Perhaps accordingly, a distinction has been made between focusing on technique and mastery, and addressing the affective aspects of being a musician; thus, Tahirbegi (2019) found a divergence between students who shared experiences of MPA with their instrumental tutors, and those who did not. Some students reported feeling comfortable discussing MPA with their teachers as they felt their teachers could meet them at an emotional level, while others said that their teachers could not offer help in MPA management or the emotional aspects of music, but were more focused on assisting them with technical and musical skills. The apparent failure of some teachers to offer emotional support is inconsistent with the emphasis on positive outlook noted previously, which evokes the value of relational qualities such as empathy, congruence, and unconditional positive regard, which might correlate better with good psychotherapy outcomes (Shaw et al., 2020).

While a range of MPA strategies had been identified across the literature, it seems clear that some educators feel ill-equipped to tackle MPA because of a lack of training or being unqualified in the area (Moura and Serra, 2021). All three of the teachers in the study by Sieger (2017) remarked that they would benefit from training in this area. Huang and Yu (2022) found that both students and teachers said that there was still a need for expert help from psychologists, psychotherapists or general practitioners. However, it is also clear that teachers can play a valuable role in this area. Studer et al. (2011) found that while 73% of the 190 undergraduate students in their study would like expert help in the field, more than half of them would like to receive this support from their university teacher. If teachers are made more aware of the overlap between teaching styles or approaches that are already embedded into regular teaching and explicit strategies designed to support MPA management, then perhaps they will feel more confident about addressing this important issue.

## 6. Limitations

The main limitation of this paper is due to the small number of articles that met the inclusion criteria. The inclusion criteria were focused on studies that investigated MPA management strategies that are used commonly in the teaching environment, rather than studies that test the transfer of specific MPA interventions in the teaching setting, of which there are numerous studies. The inclusion criteria were also focused on the role that teachers hold in this area. The small number of articles, and the fact that 6 of the 9 articles date from the last two years, indicates that this is a relatively new area of research, that is proceeding rapidly.

## 7. Conclusion

The purpose of this review was to investigate strategies used by music educators to support their students with MPA, and to explore their role in addressing MPA management with their students. A total of 96 articles were identified in the initial scoping of the literature. This was reduced to 9 articles that met the inclusion criteria.

The literature reviewed suggests that studio and classroom teachers are using multiple strategies in their lessons to support their students with MPA, with the most common being simulated performance, positive outlook, preparation, and breathing. There is evidence from the literature that there is a role for teachers to address MPA management with their students. While some students might prefer support from experts in the field of psychology, there is still demand for this support to come from the teacher. Though some teachers expressed a need for additional training in MPA management, the exploration of the literature showed that many of the strategies may be embedded in regular teaching practices to assist with the affective aspects that come with being a musician. This suggests that some of the strategies to combat MPA can be acquired from the teacher, without onerous additional training. With this understanding, perhaps supported by professional development, music educators may find that tackling the issue of MPA is more manageable under their remit. As multiple assumptions exist on whether it is the role of the teacher to manage their students MPA, this issue calls for further exploration.

## Data availability statement

The original contributions presented in the study are included in the article, further inquiries can be directed to the corresponding author.

## Author contributions

IM conducted the systematic review, analyzed the data, and wrote the drafts of the manuscript. KB and EM assisted with the analysis and editing of the manuscript. All authors contributed to the article and approved the submitted version.
